# Interactions Between Hantavirus Nucleoprotein and Glycoproteins: A Quantitative Fluorescence Microscopy Study

**DOI:** 10.3390/v17070940

**Published:** 2025-07-02

**Authors:** Amit Koikkarah Aji, Titas Mandal, Salvatore Chiantia

**Affiliations:** University of Potsdam, Institute of Biochemistry and Biology, Karl-Liebknecht-Straße 24-25, 14476 Potsdam, Germany; amitkoikkarah93@gmail.com (A.K.A.); titas.mandal@uni-potsdam.de (T.M.)

**Keywords:** protein–protein interactions, fluorescence microscopy, multimerization, virus assembly, fluorescence fluctuations, fluorescent proteins

## Abstract

Orthohantaviruses are tri-segmented negative-sense RNA viruses that can cause severe pathologies in humans. Currently, limited information exists on the molecular interactions driving orthohantavirus assembly in infected cells. Specifically, it is not clear how its glycoproteins (i.e., Gn and Gc) interact with other viral or host molecules. In this study, we use one- and two-color Number and Brightness fluorescence microscopy approaches to quantitatively characterize the interactions between orthohantavirus glycoproteins and the nucleoprotein in transfected cells. Our results indicate that orthohantavirus nucleoprotein homo-interactions are strongly affected by the host environment. Furthermore, we report evidence of Gc–nucleoprotein interactions, based on (i) the high fluorescence cross-correlation between these two proteins and (ii) the increased Gc-Gc interactions observed in the presence of nucleoprotein. Finally, experiments on a Gc deletion mutant suggest that the observed protein–protein interactions are mediated by the cytoplasmic tail of Gc. In conclusion, this study provides new insights into the role of the interactions between orthohantavirus glycoproteins and nucleoprotein in the context of viral assembly.

## 1. Introduction

**Figure 1:**
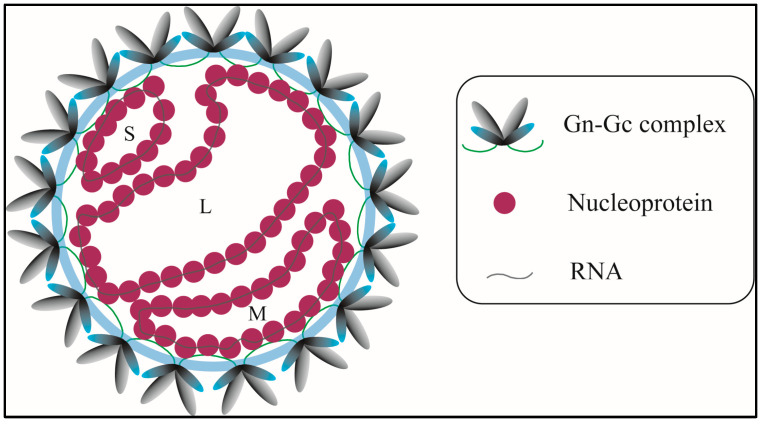
Hantavirus structure and structural proteins. The glycoproteins Gn and Gc are embedded in the lipid bilayer (light blue) and form hetero-complexes. Nucleoproteins bound to the viral RNA might interact with the cytoplasmic tails (green lines) of glycoproteins. The three RNA segments S, M, and L are labelled accordingly. Adapted from Hepojoki et al. [2].

Orthohantaviruses (HVs) (Family: Hantaviridae; Order: Bunyavirales) belong to an emerging group of zoonotic viruses that cause sporadic outbreaks around the world [1]. HVs are tri-segmented negative-sense RNA viruses: the S segment encodes the nucleocapsid protein, the M segment encodes the glycoproteins (GPs), and the L segment encodes the RNA-dependent RNA polymerase (RdRp) [2]. The interplay between HV GPs (named Gn and Gc) to form the outer envelope spike, consisting of Gn-Gc hetero-octamers, is the initial step of the viral assembly process [2,3,4]. However, limited information exists on the precise mechanisms regulating the molecular interactions involved in the subsequent stages of viral assembly. In this regard, a key event is the association between ribonucleoproteins (RNPs: comprised of viral RdRP, nucleoprotein-NP, and viral RNA) [5] and GP spikes (Figure 1). This interaction is critical for viral genome packaging and influences the progression of the viral assembly by bringing pre-assembled GP spikes into proximity, eventually leading to the formation of a new virion [2].

Our current understanding of the interaction between GP spikes and RNPs in the context of virion assembly is described by the model proposed by Hepojoki et al. [2]. This states that the cytoplasmic tail (CT) of Gn initially associates with the RNP after GP spike formation. This interaction alters the orientation of the Gn CT, thus allowing Gc to bind to the RNP via its CT. The RNP-Gn complex further regulates viral transcription and eventually promotes viral assembly. Previous biochemical studies have attempted to validate this model by primarily identifying the interacting domains between the RNP complex and GP spikes. First, the regions within the CTs of Gn and Gc responsible for the binding to NP in multiple HV strains were mapped [6,7]. Further, it was reported that the zinc finger domains in the Gn CT and the middle domain of NP are needed for NP-Gn interactions [7]. Additionally, the NP residues and nucleotides from genomic RNA that facilitate the binding of RNP to GP CTs were also characterized [8]. Finally, the observation of colocalization of Hantaan virus NP and GPs in transfected cells indicates that NP promotes Gc partition into the Golgi apparatus and that NP-Gc interactions are essential for the stabilization of the GP spike complex [9].

Despite these significant advances, some key mechanistic aspects of the proposed assembly model have not yet been explored quantitatively beyond the GP spike formation step. In this context, the molecular details of the association between NP and individual GPs are particularly relevant. HVs lack a matrix protein that mediates the interaction between the GP spike and the RNP complex and organizes the assembly of the progeny virus. The direct NP-GP association is in fact hypothesized to act as a substitute for the lack of a matrix protein [10]. However, details regarding the affinity between NP and individual GPs and the role of other viral components in stabilizing the NP-GP complex remain unclear. This is primarily due to limited quantitative information on NP-GP interactions in HV assembly, particularly within living cells. Such insights are essential for accurately understanding how the viral components are packaged to form the final structure of the nascent virion.

Here, we present a quantitative characterization of NP-GP interactions using Puumala orthohantavirus (PUUV) constructs expressed in multiple living cell models. Recently, we showed significant colocalization between fluorescently labelled PUUV NP and GPs upon co-expression of NP, Gn, and Gc [11]. However, these findings could only qualitatively suggest the presence of NP-Gc and NP-Gn interactions. In this new study, we aim to quantify NP-NP and NP-GP interactions in human or rodent epithelial cell models.

## 2. Materials and Methods

In this work, we examine NP-GP association by assessing the variations in protein multimerization and quantifying protein–protein interactions, upon co-expression of fluorescently labeled PUUV NP and GPs (Gn and Gc) in living cells. This is achieved by applying Number and Brightness (N&B) and cross-correlation N&B (ccN&B) approaches, with single-cell resolution. These methods based on the statistical analysis of fluorescence fluctuations are particularly sensitive at very low protein expression levels (between µM and nM) and can assess protein behavior in a concentration-dependent manner [12]. We have previously applied such techniques to analyze the role of specific protein–protein interactions in viral assembly [4,13].

**Cloning and generation of chimeric proteins**: The constructs used for the transcription and translation of PUUV proteins originate from the PUUV Sotkamo/V-2969/81 strain. Plasmids encoding PUUV SP-FP-Gn and PUUV SP-FP-Gc, (SP: signal peptide sequence present at the N terminus of PUUV glycoprotein precursor (GPC); FP: fluorescent protein) were previously described [4,14]. In brief, PUUV SP was introduced at the N-terminus of FP-GP (GP: Glycoprotein) to ensure physiological membrane incorporation and localization of the GP constructs [14]. The plasmid encoding PUUV YFP-NP (YFP: yellow FP) has been previously described [11]. The construct mTurqoise2 (mTurq2)-NP was designed by substituting the mEYFP of the PUUV YFP-NP plasmid with the mTurq2. The constructs SP-mEGFP-GP∆CT (mEGFP: enhanced green FP) and SP-mCh2-GP∆CT were produced by polymerase chain reaction amplification of the GP∆CT sequence (GP devoid of the cytoplasmic tail) and the subsequent product inserted between the restriction sites AgeI and Bsp1407I within the PUUV SP-FP plasmid (details on the design of PUUV FP-GP are available [4]). Gn∆CT and Gc∆CT sequences were previously described [8,15]. All sequences were verified using the Sanger sequencing facility provided by LGC (LGC, Biosearch Technologies, Berlin, Germany).

All SP-FP-GP constructs, where the FP can be mEGFP/mEYFP/mCh2/mTurq2, are referred to as FP-GP. Any deviation from this nomenclature is explicitly highlighted in the text.

**Cell Culture and transfection**: NP-GP interactions were examined in this study using different human and rodent epithelial cell models, since the pathogenesis of HV infections is influenced by the identity of the host [16]. Chinese hamster ovary (CHO) and Baby hamster kidney (BHK-21) rodent cells were chosen since they were previously used for the characterization of virus-like particle formation [17], GP multimerization [4,14], NP multimerization [18], and production of antibodies against New World HV infection [19]. Human embryonic kidney (HEK-293T) and adenocarcinomic human alveolar basal epithelial (A549) cell models were used in studies regarding host regulation factors and NP expression [20,21], cellular immunity in infection [22,23], and lentiviral expression of NP [24].

Chinese hamster ovary cells CHO-K1 (ATCC, CCL-61™), Baby hamster kidney cells BHK-21 (ATCC, CCL-10™), adenocarcinomic human alveolar basal epithelial cells A549 (ATCC, CCL-185™), and human embryonic kidney epithelial cells (HEK) from the 293T line (CRL-3216™ ) were purchased from ATCC (Kielpin Lomianki, Poland), and maintained in Dulbecco’s modified Eagle’s medium containing 10% fetal bovine serum, 100 U/mL penicillin, 0.1 mg/mL streptomycin, and 4 mM L-glutamine at 37 °C and 5% CO_2_. Cells were passaged every 3 to 5 days, no more than 15 times. All solutions, buffers, and media used for cell culture were purchased from PAN-Biotech (Aidenbach, Germany). Cells (3 to 6 × 105) were plated on glass-bottom 35 mm-diameter plates (CellVis, Mountain View, CA or MatTek Corp., Ashland, MA, USA) 48 h before experiments. Fusion protein expression plasmids were transfected into (70 to 90% confluent) CHO and HEK 293T cells using Turbofect (Thermo Fisher Scientific, Vilnius, Lithuania) according to the manufacturer’s protocol, 20 to 24 h prior to experiments. BHK-21 and A549 cells were transfected with Lipofectamine 3000, according to the manufacturer’s protocol (Thermo Fisher Scientific, Vilnius, Lithuania), 20 to 24 h prior to experiments.

**Confocal microscopy imaging**: 3 to 6 × 10^5^ cells were plated onto 35 mm glass-bottom dishes (CellVis, Mountain View, CA or MatTek Corp., Ashland, MA, USA) 48 h prior to the experiment and transfected 16 to 24 h post-seeding. Confocal images were acquired on a Zeiss LSM780 system (Carl Zeiss, Oberkochen, Germany) using a 40× DIC M27 water immersion objective. To limit the out-of-focus light, a pinhole with size corresponding to one Airy unit (~39 μm) was used. Samples were excited with a 488 nm argon laser and a 561 nm diode laser. The fluorescence signal was collected by a Zeiss QUASAR multichannel GaAsP detector in photon-counting mode. The laser power was set so that the photon count rate and bleaching remained below 1 MHz and ca. 20%, respectively (typically ~3 μW for 488 nm, and ~5 µW for 561 nm). Measurements exhibiting stronger bleaching were discarded. Fluorescence was detected between 499 and 552 nm (mEGFP) and between 570 and 695 nm (mCh2), after passing through an MBS 488/561 dichroic mirror.

**Number and Brightness (N&B)**: Number and Brightness analysis was performed as previously described [4]. Briefly, images of 128 × 128 pixels were acquired with pixel dimensions of 400 nm and a pixel dwell time between 25 and 50 µs. Image time-stacks of 100 scans were collected using the Zeiss Blu ZEN 3.8 software. The intensity time-stacks data were imported into MATLAB 2022 using the Bioformat [25] package and analyzed using a custom-written code (The MathWorks, Natick, MA, USA). The algorithm uses the equations from [26] for the specific case of photon-counting detectors to obtain the molecular brightness and number as a function of pixel position. Upon selecting the required region of interest (ROI), fluorophore bleaching and minor cell movements within a few pixels are suitably corrected. ROIs purposedly did not include any immobile structures, such as large aggregates or immobile protein filaments. Pixel intensities are then used to calculate the average brightness of the FP detected within the cellular ROI. This average brightness is utilized to evaluate the multimerization state of the FP upon comparison to a reference monomer brightness [27]. Thus, the oligomeric size of the protein complex is evaluated in terms of monomer units.

**Cross Correlation Number and Brightness (ccN&B)**: The ccN&B analysis is based on a published workflow [13], modified suitably for the evaluation within the cytosolic region of the cell. Briefly, an image stack is acquired over time typically consisting of 100 frames. Images of 256 × (y< 256) pixels with a pixel size of 0.21 µm and a pixel dwell time of 12.6 µs were acquired, alternating two different excitation wavelengths. CZI-format image output files were imported into MATLAB using the Bioformat [25] package and analyzed using a self-written script implementing the framework described by Digman et al. [28] for the specific case of photon-counting detectors and two-color excitation. Pixels corresponding to a ROI are manually selected in an image map. Next, to account for lateral drift during the acquisition, frames are aligned to the first frame by maximizing the spatial correlation between sub-selections in consecutive frames, averaged over both channels, as a function of arbitrary translations. Additional steps regarding corrections for bleaching, minor cell movements, and specific detector response are applied as previously described [13].

The ccN&B analysis calculates the dimensionless parameter relative cross correlation (Relative CC). Relative CC is calculated as [13]:

Relative CC = *max* (N_cc_/N_g_, N_cc_/N_r_)

Here, N_g_ and N_r_ are the particle numbers detected in the image channel corresponding to the green FP and red FP, respectively. N_cc_ is obtained by dividing the product of the average intensities of the green and red FP channels by the covariance of the two channels.

**Statistical Analysis**: The statistical analysis in this work was performed using GraphPad Prism version 9.0.0 (GraphPad Software, LCC, San Diego, CA, USA). For pairwise comparisons of the derived parameters, the Kruskal–Wallis Test and modified Tukey’s multiple comparisons test were used.

## 3. Results

### 3.1. NP-NP Interactions in Human Epithelial Cells Are Stronger than in Rodent Epithelial Cells

Hägele et al. [29] observed significant variations in NP localization and function between infected Vero E6 cells and primary human renal cells. This allowed them to conclude that the behavior of NP might depend on the specific cell type. Here, we aimed to determine whether different host environments (namely, human vs. rodent epithelial cell models) might influence homotypic NP-NP interactions in transfected cells.

NP multimerization in cells imaged via confocal microscopy was examined using N&B analysis, a quantitative approach based on the statistical fluctuations of the fluorescence signal within the probed confocal volume. This approach was previously used to monitor NP multimerization in CHO cells [11]. In this work, we extend the analysis to compare the average NP multimerization detected in human epithelial cells (HEK and A549) and rodent epithelial cell models (BHK-21) 16 to 20 hpt.

**Figure 2:**
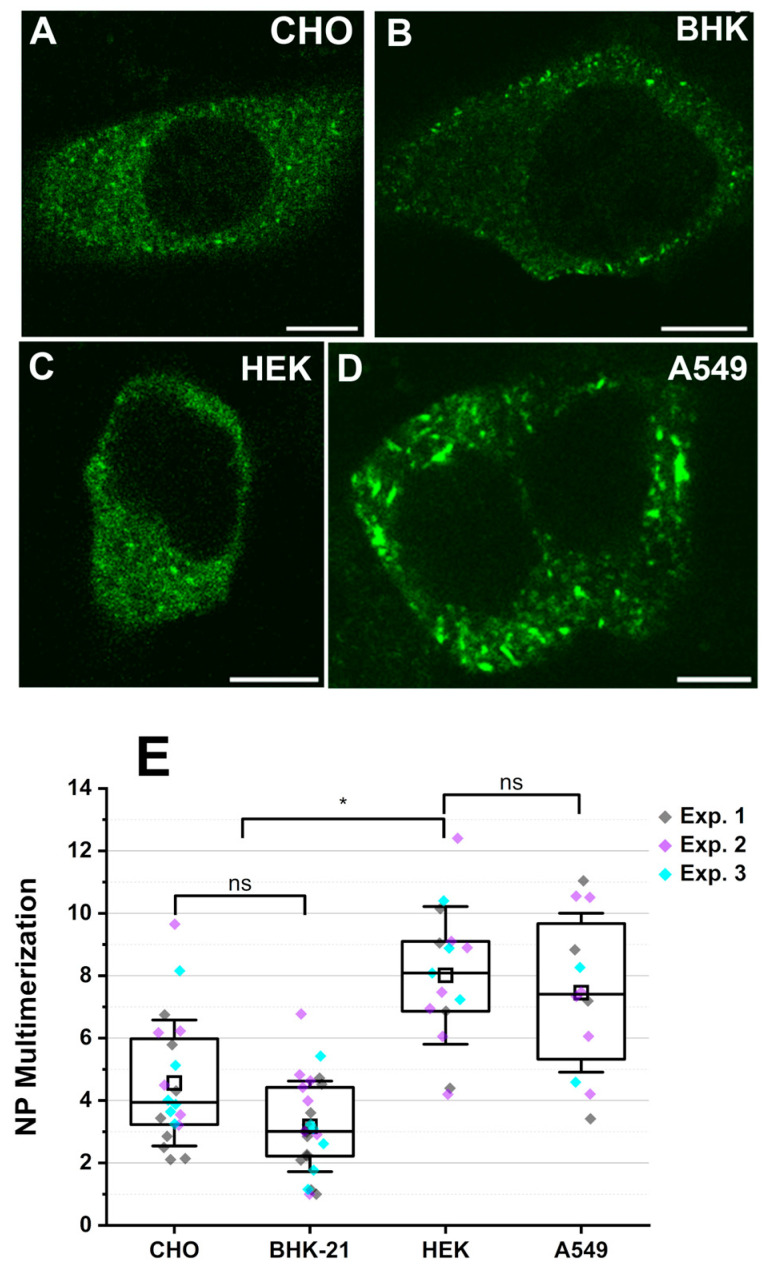
Average multimerization of fluorescently labelled PUUV NP is higher in human cell models compared to rodent epithelial cell models. YFP-NP was expressed in multiple epithelial cell models and observed 16 to 20 hpt. Panels (**A**–**D**) show representative confocal microscopy images of (**A**) CHO, (**B**) BHK-21, (**C**) HEK 293T, and (**D**) A549 cells expressing YFP-NP. Panel (**E**) shows a quantitative comparison of YFP-NP multimerization between different cell models. For this analysis, cells expressing YFP-NP within the concentration range 0.1–0.5 µM were chosen. Data points for NP multimerization in CHO cells are from Welke et al. [11] and incorporated as reference values. Each point in the boxplot represents the average multimerization in one cell. At least 12 cells were analyzed for each case, with three technical replicates denoted in different colors (see Supplementary Information for quantitative details). In all box plots, the horizontal line is the median, ‘□’ marks the mean, with the first and third quartile as the boundaries and whiskers as standard deviation (SD). Statistical analysis was performed using the pairwise Kruskal–Wallis one-way ANOVA test (ns: not significant, * *p* < 0.01). Scale bars are 10 µm.

As previously shown by Davies et al. [30], NP initially appears homogenously distributed in the cytoplasm. This is followed by the formation of small mobile clusters, for all cell types tested (Figure 2A–D), ca. 4 hours post-transfection. At later time points (i.e. after ca. 12 to 16 hours), NP starts to coalesce, forming large immobile filaments [11], but such samples were not included in the current analysis.

According to N&B analysis, NP forms small oligomers, up to ca. decamers on average, for all cell models tested, within the entire concentration regime explored (Figure S1). More specifically, NP multimerization values in BHK cells increase strongly with protein concentration, as previously observed for CHO cells [11] (shown in Figure S1, as reference). On the other hand, multimerization values measured in A549 and HEK cells are higher (especially at lower concentrations) and display a less marked correlation with protein concentration (Figure S1). Since different cell models displayed variability in the expression levels, we compared the average NP multimerization in human and rodent cell models within a restricted concentration range (0.1 to 0.5 µM): within this concentration range, we were able to collect a similar amount of data points for all cell models and, therefore, a direct comparison of average values is reasonable. In these conditions, NP forms on average tetramers in rodent epithelial cells and roughly octamers in human epithelial cells (Figure 2E).

We further attempted a statistical analysis of the multimerization–concentration curves, for different cell models and across the whole available protein concentration range, by fitting an empirical model to the data [31], as shown in Figure S1. It is worth noting that such a fitting is not meant to deliver precise parameters, but rather to allow a statistical comparison of the different datasets. In agreement with the above-mentioned observations, NP multimerization behavior in rodent (CHO [11] and BHK-21) and human epithelial cell models (HEK-293T and A549) appears to be significantly different over the observed concentration range (Supplementary Table S1).

Altogether, these results indicate that the average NP-NP interactions observed in human epithelial cells are stronger than in rodent epithelial cell models, at least at the early stages of NP expression and assembly.

### 3.2. NP Interacts with Gc Within Few Small Intracellular Regions

We proceeded to compare the interactions between PUUV NP and each PUUV GP (Gn or Gc) upon co-expression in different human (HEK 293T and A549) and rodent epithelial (CHO and BHK-21) cell models. Welke et al. [11] previously indicated that NP, Gn, and Gc displayed significant spatial colocalization in transfected CHO cells. While these findings indicate that NP and GPs are found in close vicinity to each other, they do not directly imply an interaction between the proteins. Therefore, we performed two-color ccN&B analysis to investigate whether NP significantly interacts with any of the GPs. This method provides a parameter (i.e., relative CC) that indicates the relative amounts of co-diffusing hetero complexes.

**Figure 3:**
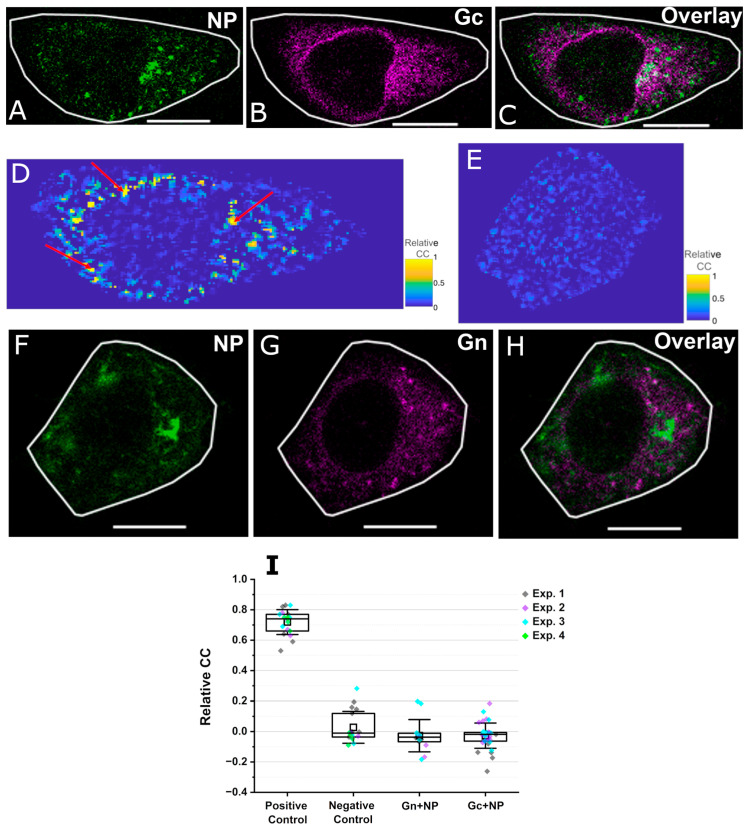
Single-cell analysis and spatial maps of the interactions between NP and GPs. Panels (**A**–**C**) show representative images of CHO cells co-expressing PUUV YFP-NP and PUUV mCh2-Gc, 20 to 24 hpt. Panel (**D**) shows the pixel-by-pixel relative CC map of the same CHO cell, within a ROI as indicated by a white line in panels (**A**–**C**). Red arrows in panel (**D**) denote localized regions characterized by high relative CC values. Panel (**E**) shows the pixel-by-pixel relative CC map of the same CHO cell, within a ROI as indicated by a white line in panels (**F**–**H**). Panels (**F**–**H**) show representative images of CHO cells co-expressing PUUV YFP-NP and PUUV mCh2-Gn, 20 to 24 hpt. Panel (**I**) shows a box plot of the relative CC values averaged over whole-cell ROIs and measured for different constructs expressed in CHO cells. Positive control is a tandem YFP-mCh2 construct that localizes in the cytoplasm. The negative control refers to cells co-expressing YFP-NP and cytosolic mCherry2. ROIs in cells with intensity values less than 1 MHz in the YFP and mCherry2 channel were chosen for evaluating the average relative CC within each cell. Each point in panel (**I**) represents the average relative CC value from one ROI in one cell. Number of points for each case >15, from three independent experiments denoted in individual colors (see Supplementary Information for quantitative details). In all box plots, the horizontal line is the median, ‘□’ marks the mean, with first and third quartile as the boundaries and whiskers as SD. Scale bars are 5 µm.

Figure 3A–C and Figure 3F–H show representative confocal microscopy images of cells co-expressing YFP-NP and either mCh2-Gc or mCh2-Gn in CHO cells, respectively. The intracellular localization of Gn and Gc within the ER and/or perinuclear region is similar to what was previously observed [4].

To quantify hetero-interactions, ccN&B analysis was performed, thus obtaining relative CC maps, as shown in Figure 3D and E for NP-Gc and NP-Gn interactions, respectively. Additional examples of relative CC maps are shown in Figure S2I,J. Overall protein–protein interactions were first quantified by averaging the relative CC from each pixel in the selected ROIs (roughly corresponding to the whole intra-cellular region). As shown in Figure 3I for CHO cells, significant interactions were detected for the positive control (i.e., a tandem cytosolic construct of YFP and mCh2) but not for the negative control (consisting of independently co-expressed YFP-NP and cytosolic mCh2). Similar results were obtained for the controls in all cell models tested (Figure S3). The interactions between NP and either GP (i.e., Gn or Gc), averaged over the whole cell, were relatively weak for all cell models tested (CHO cells, see Figure 3I; HEK cells, see Figure S3A; and A549 cells, see Figure S3B) and not distinguishable from the negative control. Co-expression of fluorescently labelled NP and GP constructs in BHK-21 cells did not yield a GP expression high enough for quantifying relative CC and, therefore, this cell model was not used for subsequent analysis.

Next, we analyzed in greater detail the spatial variations of the relative CC parameter in cells expressing the different constructs. As expected, CHO cells expressing the tandem mEYFP-mCh2 construct (positive control) exhibited positive relative CC values homogenously distributed throughout the analyzed intracellular ROI (Figure S2E–H). Similarly, the analysis of CHO cells expressing YFP-NP and cytosolic mCherry2 (negative control) resulted in homogeneous low relative CC values throughout the cell (Figure S2A–D). Interestingly, similarly to the negative control, we observed relatively homogeneous intracellular distribution of low relative CC values for cells co-expressing Gn and NP (Figure 3E and Figure S2I). On the other hand, we consistently noticed localized regions characterized by high relative CC values (i.e., high local abundance of co-diffusing Gc-NP complexes) in CHO cells co-expressing Gc and NP (see red arrows in Figure 3D and Figure S2J). Such regions were mostly observed around the perinuclear region and, most importantly, did not display an evident correlation with the spatial distribution of either Gc or NP (see Figure 3A,B,D). 

Altogether, these results demonstrate that PUUV GPs interact, on average, weakly with PUUV NP in intracellular regions, for all cell models tested. Nevertheless, PUUV Gc appears to interact significantly with PUUV NP within localized intracellular regions close to the perinuclear region.

### 3.3. Apparent Gc Multimerization Increases in the Presence of NP

The experiments described above suggest that NP might play a role in intermolecular interactions involving Gc, but not Gn. To further explore this interaction network, we measured GP-GP interactions (i.e. the multimerization state of each GP) in the presence of NP, using single-color N&B in different cell models. As for the ccN&B analysis, cells displaying a quasi-homogeneous distribution of NP within the cytoplasm (see Figure 2A–D) were chosen.

**Figure 4:**
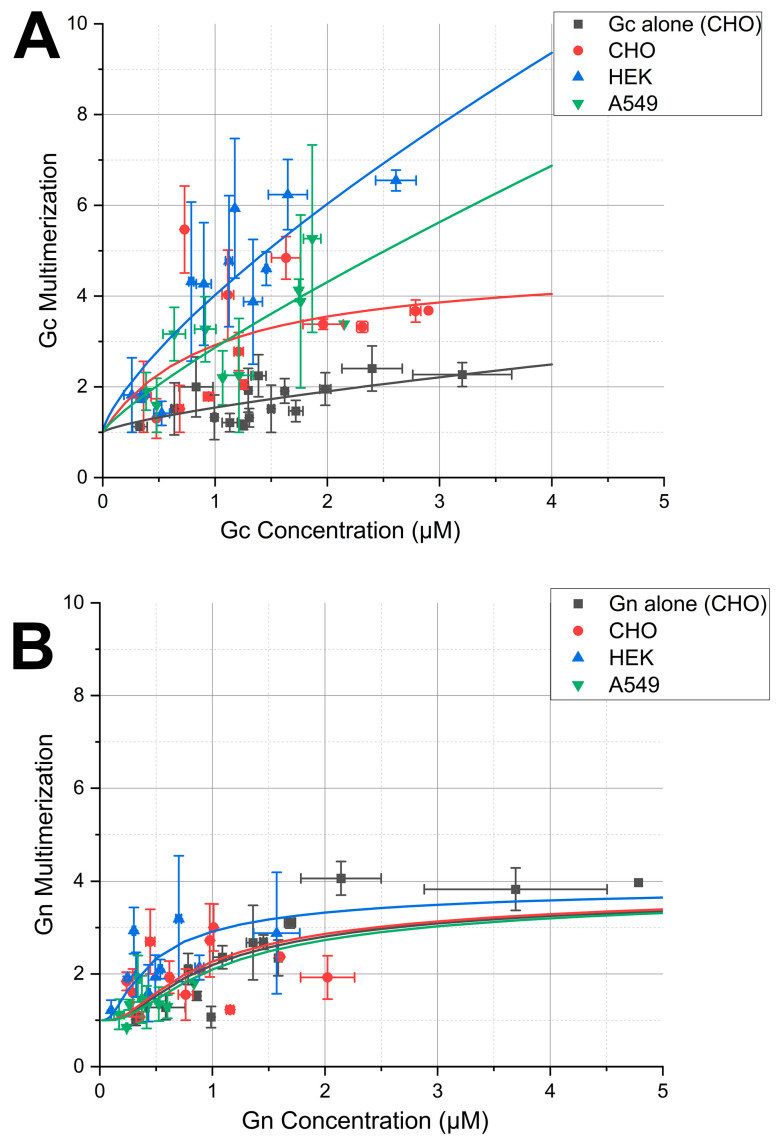
PUUV Gc (but not Gn) multimerization behavior is altered in the presence of NP. Panel (**A**) shows the concentration-dependent multimerization analysis of PUUV Gc in the presence of PUUV NP using N&B, in different epithelial cell models: CHO (red), A549 (green), and HEK 293T (dark green). PUUV Gc multimerization in the presence of PUUV NP is compared to that measured in the absence of NP in CHO cells (black, data from [4]). Each point in the graph represents the binned average multimerization values from two cells. The solid lines represent a fit to an empirical power growth model [31] in the form of y = 1 + a∙x^k^. Fit results and statistical analysis are shown in Table S2. Panel (**B**) shows the concentration-dependent multimerization analysis of PUUV Gn in the presence of PUUV NP using N&B, in the same epithelial cell models as for panel A. PUUV Gn multimerization in the presence of PUUV NP is compared to that measured in the absence of NP in CHO cells (black, data from [4]). Each point in the graph represents the binned average multimerization values from two cells. The solid lines represent a fit to a monomer–tetramer equilibrium model [32]. Fit results and statistical analysis are shown in Table S3.

PUUV Gc multimerization in the presence of PUUV NP was analyzed by co-expressing YFP-NP and mCh2-Gc in CHO (Figure 3A–C), HEK (Figure S4A–C), and A549 (Figure S4D–F) cells and performing N&B measurements 24 hpt. Our previous work shows that the expression of Gc alone results in a mixture of monomers and dimers in the ER of different cell models [4,14]. Strikingly, in the presence of NP, Gc forms large multimers (ca. up to octamers) instead, for all the cell models tested, within the explored concentration range (Figure 4A). Thus, Gc-Gc interactions in the presence of NP are significantly stronger than those previously observed in the absence of NP (shown in black in Figure 4A as reference, [4]).

To compare different datasets, the multimerization curves were analyzed using a simple empirical growth model with two free parameters [32]. The resulting curves for all the examined cells expressing both Gc and NP lay considerably above the multimerization curve obtained in CHO cells expressing only Gc (*p* < 0.01), thereby confirming the initial qualitative observation. Only minor differences can be observed between the other curves, mostly due to the large data spread (see Table S2).

### 3.4. Gn Remains in a Monomer–Tetramer Equilibrium, Independent of the Presence of NP

We further examined the multimerization of Gn in the presence of NP. CHO (Figure 3E–G), HEK (Figure S5A–C), and A549 (Figure S5D–F) cells co-expressing fluorescently labelled Gn and NP were observed 20 to 24 hpt. Cells/ROIs in which NP formed homogeneously distributed small mobile oligomers were selected, thus excluding from the analysis large immobile NP structures. We have previously shown that Gn forms up to tetramers in the absence of other viral proteins [4]. The multimerization values obtained in this work (Figure 4B) in the presence of NP are compatible with similar multimerization behavior, for all the cell models tested, at least within the restricted concentration range that could be explored.

To quantitatively compare the different datasets, multimerization curves were fitted to an analytical model that describes a monomer to tetramer equilibrium through the association constant K_4_ [32]. This parameter was not statistically distinguishable among the different samples (0.2 < *p* < 0.8) (see Table S3). This result indicates that Gn-Gn interactions are not strongly affected by the presence of NP.

### 3.5. Deletion of Gc-CT Domain Abolishes the Enhancement of Gc-Gc Interactions Induced by NP

**Figure 5:**
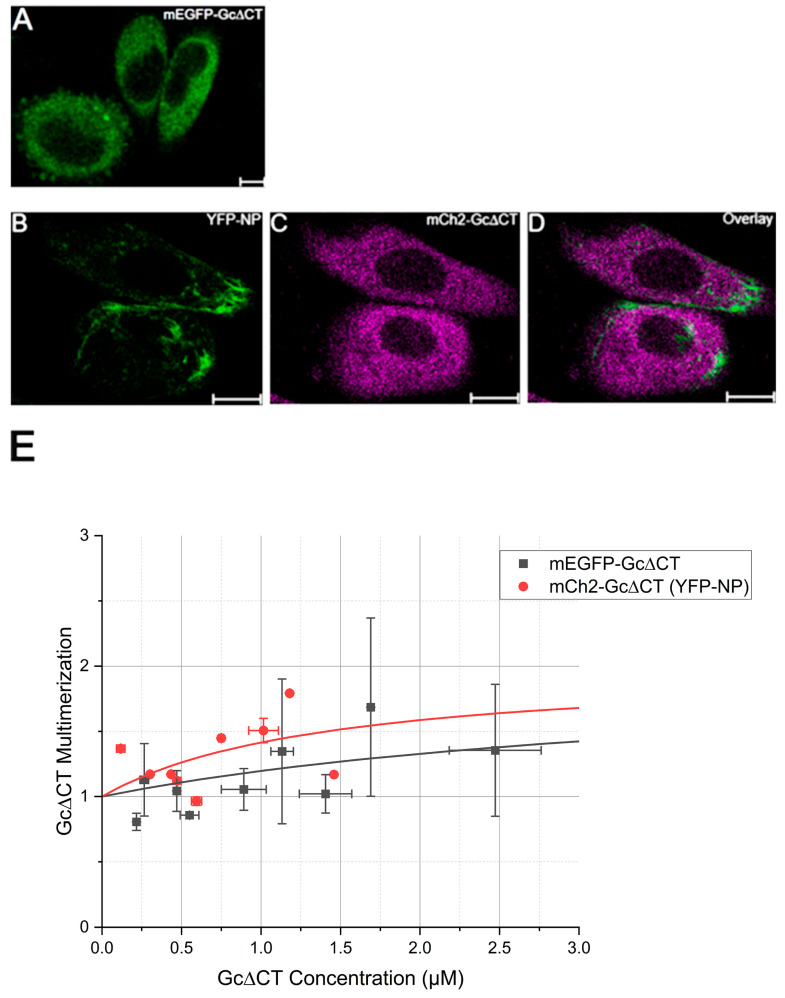
Deletion of Gc∆CT inhibits the formation of large Gc assemblies in the presence of NP. Panel (**A**) shows a representative image of CHO cells expressing PUUV mEGFP-Gc∆CT and observed 24 hpt. Panels (**B**–**D**) show typical confocal image of CHO cells co-expressing YFP-NP and mCh2-Gc∆CT. Panel (**E**) shows the concentration-dependent multimerization analysis of PUUV Gc∆CT in the absence (black) and presence (red) of PUUV NP, calculated using N&B analysis. Each point in the graph represents the binned average multimerization from two cells. The solid lines represent a fit to a monomer–dimer equilibrium model [32]. Fit results and statistical analysis are shown in Table S4. Scale bars are 10 µm.

Finally, the influence of the GP-CT domain on GP multimerization in the presence of NP was examined, since NP was proposed to interact with GPs via their CTs [6,7]. Truncated GPs without CT domains (GP∆CT) were initially expressed in cells in the presence and absence of NP (Figure 5A–D for Gc∆CT, Figure S6A–D for Gn∆CT). The intracellular distribution of the GPs does not appear to be strongly influenced by the presence of NP (Figure 5A,C and Figure S6). Also, the spatial organization of NP is similar to what was previously observed 24 hpt in CHO cells in the absence of other viral proteins [11].

We thus proceeded to analyze GP∆CT multimerization 20 to 24 hpt. Figure 5E shows that fluorescently labelled Gc∆CT, in the absence of NP, displays an average multimerization of ~1.4 at the highest measured concentration. According to this value and assuming a monomer–dimer equilibrium, the protein appears to form mostly monomers (ca. 75 mol%), in agreement with previous measurements on non-fluorescent constructs in vitro [33]. In the presence of YFP-NP, the multimerization of Gc∆CT did not change substantially (Figure 5E). Similar results were obtained with A549 cells (Figure S7). The concentration-dependent multimerization data points in Figure 5E were analyzed with an analytic model describing monomer–dimer equilibrium [32]. The derived association constant K_2_ did not change significantly in the presence or absence of NP (Table S4).

The influence of Gn-CT on Gn-Gn interactions was similarly analyzed. Figure S6E shows that fluorescently labelled Gn∆CT, in the absence of NP, displays an average multimerization value compatible with the presence of a large number of monomers in equilibrium with dimers, in agreement with the results obtained using non-fluorescent constructs [17]. In the presence of YFP-NP, the average multimerization of Gn∆CT remained similar. Analogous results were obtained with A549 cells (Figure S8).

In summary, the increased formation of Gc-Gc complexes induced by NP appears to be mediated by the Gc-CT domain, for all the cell models tested. On the other hand, we did not observe any influence of NP on the multimerization of Gn, irrespective of the presence of its CT.

## 4. Discussion

NP is a multifunctional HV protein that is capable of interacting with different host proteins and other viral components and mediates the association between the RNP and GPs [34]. While previous studies have focused on the protein regions possibly involved in the binding between NP and GPs, there is limited information regarding the molecular details of the actual interactions between NP and either Gc or Gn occurring in vivo. In this study, we address this important question by quantifying the interaction between NP and GPs upon co-expression, at the single-cell level. In order to pinpoint specific interactions between NP and Gn or Gc, we evaluated the behavior of these proteins in the absence of other viral components (e.g. viral RNA) in live cell models, through transient transfection.

HV infections display distinct cellular tropism [16,22,29]. Therefore, we initially assessed if a specific cellular environment could influence NP-NP binding, by comparing human and rodent epithelial cell models. We observed that average homotypic NP-NP interactions are significantly stronger in human epithelial cell models compared to rodent epithelial cell models, at a given time point post-transfection. This observation is in line with previous studies [29] that reported point-like NP structures in infected Vero E6 cells, smaller than the filamentous NP assemblies present instead in infected human renal cells. Also, it has been shown that cytoskeletal components, such as actin, vimentin, and microtubules, influence NP organization in a fashion that varies among different HV strains and cell models [29,35]. It is therefore possible that the modulation of NP-NP interactions described in this study may be determined by cell type-specific interactions with cytoskeletal components. Alternatively, post-translational modifications, including phosphorylation [36,37] or SUMOylation [38] may also contribute to the regulation of NP multimerization in a cell type-dependent manner. A third possibility is that NP multimerization is influenced by other host-specific factors [39], e.g., proteins binding to the NP multimerization interface. Independent from the specific molecular mechanism, it would be very interesting to investigate whether alterations in NP multimerization equilibrium are truly correlated to the susceptibility of a specific host cell to HV infection.

In the second part of our investigation, we observed weak overall NP-GP interactions, independent of the tested cell model, while NP still mostly formed small mobile clusters. Interestingly, within single cells, localized regions were detected in which NP showed an apparently strong interaction with Gc. We are currently investigating whether locally enhanced NP-Gc interactions might exhibit a spatial correlation with, e.g., cytoskeletal elements or other intracellular structures. Interestingly, such local interactions with NP were never observed for Gn. Previous studies have shown significant interactions between these two proteins via the Gn CT domain in infected cells using co-immunoprecipitation [6]. Nevertheless, it must be noted that these experiments were performed several days post-infection, i.e., at a time point in which NP forms large immobile structures [30] and in the presence of other viral components. This observation suggests that NP-Gn interactions might possibly be mediated by viral RNA [6,8]. Also, our results additionally show that the quantitative microscopy approach with single-cell resolution described here can provide more detailed information, compared to bulk methods that average over a large number of cells.

While the fluorescence fluctuation microscopy analysis used in this work (N&B) delivers quantitative information about inter-molecular interactions and has a high spatial resolution, it also requires the observed molecules to be mobile during the observation. Although we restricted our analysis to cells which did not show large immobile structures, it is known that NP might form a small fraction of larger very slow NP aggregates, as described by Welke et al. [11]. If the reported higher CC values (i.e. stronger interactions) do indeed originate from such almost immobile NP fractions, NP-GP interactions cannot be quantified precisely with this approach. For this reason, we complemented these measurements by separately observing GP multimerization in the presence of NP.

In the case of Gc, we observed a striking difference in homotypic interactions when NP is also present. While not a direct measurement of NP-Gc interaction, this is compatible with the above-mentioned results, under the hypothesis that the observed Gc multimer formation is indeed somehow mediated by interactions with NP (e.g., through the binding of several individual Gc molecules to a large NP multimer). Gc localization within NP clusters was also previously observed for other Bunyaviruses, such as Tomato spotted wilt virus [40,41].

Furthermore, our experiments on truncated Gc constructs indicate that NP-Gc interaction occurs via the Gc CT domain, as also suggested by previous studies [2,9]. This result strengthens the hypothesis that Gc CT can act analogously to a matrix protein [10], connecting the envelope of the virus with the enclosed genetic material. Such interactions might play a role in the large-scale assembly of the GP spikes during virus assembly and confer mechanical stability to the new virions.

Alternatively, it is also possible that the removal of the CT affects the stability and structure of Gc [14] and, thus, its interaction with NP. Taken together, our findings suggest that HV NP can form complexes with Gc (possibly including other molecules as well), also in the absence of Gn or RNPs.

On the other hand, the lack of direct interactions between Gn and NP as observed via ccN&B is corroborated by the fact that, in the observed cell models, Gn multimerization is independent of the presence of NP, with this GP consistently forming monomers to tetramers, as expected.

In conclusion, we have investigated here the interactions between HV NP and GPs using quantitative fluorescence microscopy approaches. According to N&B experiments, NP can interact with Gc in the absence of other viral components, but not with Gn. The observed interactions appear to be mediated by the CT of Gc. Notably, all the experiments performed in this work involve the transient expression of up to two viral proteins at the same time, with the purpose of specifically identifying direct interactions (i.e., not involving other viral proteins). As mentioned above, a more comprehensive picture of the interaction network can be obtained if additional viral components are systematically added to the studied system (e.g. RNA to further characterize NP-Gn interactions). Future studies will, for example, focus on quantifying interactions between fluorescently labelled NP, Gn, and Gc, also using third-order correlation functions [42]. Such experiments are complicated by the interference of fluorescent labels on inter-molecular interactions (e.g. between Gn and Gc) [4]. By using more advanced methodologies for labelling (see, e.g., ALFA tag [43], bioorthogonal click chemistry [44]), the approach described in this work could open new avenues for the precise characterization of binding between other HV proteins and further elucidate the steps involved in the HV virion formation.

## Data Availability

The original contributions presented in this study are included in the Supplementary Material. Further inquiries can be directed to the corresponding author.

## Supplementary Materials

The following supporting information can be downloaded at: https://www.mdpi.com/article/10.3390/v17070940/s1. Figure S1: Homotypic NP-NP interactions differ between human and rodent epithelial cell models; Table S1: Statistical analysis of the fit results deriving from the comparison of NP multimerization between cell models; Figure S2: Intensity and relative CC maps of CHO cells expressing positive and negative control; Figure S3: Average interactions between NP and GPs co-expressed in HEK and A549 cells are weak; Figure S4: Co-expression of PUUV NP and PUUV Gc in HEK293T and A549 cells; Table S2: Statistical comparison of fit parameters describing Gc multimerization between cell models co-expressing NP and Gc; Figure S5: Co-expression of PUUV NP and PUUV Gn in HEK293T and A549 cells; Table S3: Statistical comparison of fit parameters describing Gn multimerization between cell models co-expressing NP and Gn; Table S4: Statistical comparison of fit parameter describing Gc∆CT multimerization between cell models co-expressing NP and Gc∆CT; Figure S6: PUUV Gn∆CT remains mostly monomeric, independent of the presence of NP, upon expression in CHO cells; Figure S7: Lack of Gc∆CT inhibits the formation of large Gc assemblies in the presence of NP, also in A549 cells; Figure S8: PUUV Gn∆CT is mostly monomeric, independent of the presence of NP, upon expression in A549 cells; Table S5: Statistical parameters of the data shown in Figure 2 of the main manuscript; Table S6: Statistical parameters of the data shown in Figure 3 of the main manuscript. Numerical data and raw data (i.e., before binning) for all figures are provided in the form of two spreadsheets.

## Abbreviations

The following abbreviations are used in this manuscript:

HVs	Orthohantaviruses
GPs	Glycoproteins
RdRp	RNA-dependent RNA polymerase
RNPs	Ribonucleoproteins
NP	Nucleoprotein
CT	Cytoplasmic tail
PUUV	Puumala orthohantavirus
N&B	Number and Brightness
ccN&B	Cross-correlation N&B
CHO	Chinese hamster ovary
HEK	Human embryonic kidney
A549	Adenocarcinomic human alveolar basal epithelial
SP	Signal peptide
GPC	Glycoprotein precursor
FP	Fluorescent protein
YFP	Yellow fluorescent protein
mTurq2	Monomeric Turqoise2
mEGFP	Monomeric enhanced green FP
∆CT	devoid of cytoplasmic tail
SD	standard deviation
